# Healthy Men and Women

**Published:** 1871-09

**Authors:** 


### HEALTHY MEN AND WOMEN.

It is the stock in trade of many who write for the papers to make sweeping assertions about the unhealthy condition of women as a class. But what about the men? With their drinking, and smoking, and late hours, and clubs, and haunts still lower, it has come to pass that in meeting any ten men on any street, there shall not be two who will pass the muster of perfect health. The truth of this is demonstrated in the fact which cannot be denied, that there are more women than men; there are more old women than old men. And when men do grow very old, they are too often useless; they are in the way; they cannot do anything about the house, they are so awkward, so clumsy. An old woman of the same age can make herself useful in many ways. She can help about the house, she can sympathize, she can encourage, she can hold up. An old man is fit for nothing but to loll on the sofa, sit around the fire, talk politics, and too often prone to grunt and groan and find fault with everything and everybody but himself. He is always in the way. It is a great pity that some of them could not be laid up on shelves, like an old magazine or newspaper. Alas for the old men; those of them who are poor, and have outlived their wives and children. While Lenox and Saunders spend fortunes in founding " Homes " for respectable old women, and A. T. Stewart gives his millions to provide comfortable homes for sewing girls, and cheap homes in the country for city clerks, it is to be hoped that some philanthropist will soon arise to build a home for the poor, friendless old man.

